# Cost of antipsychotic polypharmacy in the treatment of schizophrenia

**DOI:** 10.1186/1471-244X-8-19

**Published:** 2008-04-04

**Authors:** Baojin Zhu, Haya Ascher-Svanum, Douglas E Faries, Christoph U Correll, John M Kane

**Affiliations:** 1 Eli Lilly and Company, Indianapolis, Indiana, USA; 2 The Zucker Hillside Hospital, Glen Oaks, New York, USA

## Abstract

**Background:**

This study compared the costs of antipsychotic polypharmacy for patients who initiated on 1 of the 3 most commonly prescribed atypical antipsychotics – olanzapine, quetiapine, or risperidone.

**Methods:**

Data were drawn from a large, prospective, naturalistic, multi-site, nonrandomized study of treatment for schizophrenia in the United States conducted between July 1997 and September 2003. Participants who were initiated on olanzapine (N = 405), quetiapine (N = 115), or risperidone (N = 276) were followed for 1 year post initiation and compared on: (a) average daily cost of the index antipsychotic while on the index antipsychotic, (b) average daily cost of the coprescribed antipsychotics while on the index antipsychotic, (c) average daily cost of the index antipsychotic and the coprescribed antipsychotics while on the index antipsychotic, (d) total annual cost of antipsychotic medications prescribed in the year following initiation on the index antipsychotic, using propensity score-adjusted bootstrap resampling method. Average daily antipsychotic costs and total annual antipsychotic costs were also estimated using more recent (2004) antipsychotic drug prices.

**Results:**

During the 1 year following initiation on the index antipsychotic, the total average daily cost of the index antipsychotic was higher for quetiapine ($15.33) than olanzapine ($13.90, p < .05) and risperidone ($11.04, p < .01), although the average daily cost of the index antipsychotic was higher for olanzapine ($10.08) than risperidone ($6.74, p < .01) or quetiapine ($6.63, p < .01). Lower total average daily costs were observed in risperidone than olanzapine or quetiapine. Significantly lower average daily cost of concomitant antipsychotic medications for olanzapine ($3.82) compared to quetiapine ($8.70, p < .01) or risperidone-initiated patients ($4.30, p < .01) contributed to the lower average daily cost of all antipsychotic medication for olanzapine-initiated patients. Each dollar spent on the index antipsychotic was accompanied by spending an additional $1.31 on concomitant antipsychotics for quetiapine compared to $0.64 for risperidone and $0.38 for olanzapine-initiated patients. A separate intent-to-treat analysis of the total annual antipsychotic cost found a significantly higher total annual antipsychotic cost for quetiapine-initiated patients ($5320) compared to olanzapine ($4536, p < .01) or risperidone ($3813, p < .01).

**Conclusion:**

Prevalent antipsychotic polypharmacy adds substantial cost to the treatment of schizophrenia. Comparison of medication costs need to address the costs of all antipsychotics. A better understanding of concomitant antipsychotic costs provides a more accurate portrayal of antipsychotic medication costs in the treatment of schizophrenia.

## Background

Antipsychotic medications have been a core treatment modality in the treatment of patients with schizophrenia over the past 40 years with antipsychotic monotherapy being the treatment of choice [1]. Despite consistent recommendations of antipsychotic monotherapy, antipsychotic polypharmacy is commonplace in the treatment of schizophrenia [2-9]. Polypharmacy – the concomitant use of 2 or more antipsychotics – has been reported to range between 13% and 60% depending on the population studied, the year the study was conducted, the study method, the type of treatment site, and the duration of the study period [7,9-14]. Our recent study using data from an observational noninterventional study [15] found that over a 1-year study period only a third of the schizophrenia patients were treated predominantly with antipsychotic monotherapy, whereas 58% of schizophrenia patients had at least 1 period of antipsychotic polypharmacy lasting longer than 60 consecutive days.

In recent years as additional atypical antipsychotics with different pharmacological properties have become available for the treatment of schizophrenia, the likelihood of combining antipsychotics has increased [16,17]. The rate and duration of antipsychotic polypharmacy was previously shown to significantly differ among atypical antipsychotics [5,9], as olanzapine-initiated patients were often found more likely to be treated on monotherapy while quetiapine-initiated patients were more likely to be treated with polypharmacy [10,13,15,18-23].

While the clinical benefits of antipsychotic polypharmacy are unclear and poorly documented [5,9,24-26], the use of polypharmacy may also hinder clinicians' ability to accurately evaluate a patient's response to a new course of treatment [2]. It also increases the complexity of the medication regimen and risk of adverse events and makes it more difficult to assess and manage future symptom exacerbations [5]. Moreover, polypharmacy increases antipsychotic treatment costs at a time of growing budget constraints across systems of care [5,8,19]. Despite concerns about economic consequences of antipsychotic polypharmacy, most recent studies on antipsychotic medication costs have focused mainly on index atypical drug acquisition costs, and no information is yet available on differential cost of polypharmacy during the usual care of schizophrenia with commonly prescribed atypical antipsychotics.

To fill the gap in knowledge and expand on previous findings, we used data from a large, prospective, noninterventional, observational study in the United States to assess the cost of antipsychotic polypharmacy during the long-term treatment of schizophrenia patients in usual care. We have calculated the average daily cost of each "index" antipsychotic (olanzapine, risperidone, and quetiapine) and the average daily cost of the coprescribed antipsychotics by medication type (typical and atypical). In addition to average daily costs, this study also used an intent-to-treat approach to assess the total annual costs of all antipsychotic medications prescribed in the year following initiation on each index atypical antipsychotic. To enhance relevance of the findings, the average daily and total annual costs of antipsychotic polypharmacy were also estimated using more recent (2004) drug prices.

## Methods

### Data source and sample selection

This study used data from the U.S. Schizophrenia Care and Assessment Program (US-SCAP), a large, nonrandomized, naturalistic, 3-year prospective, multi-site study conducted between July 1997 and September 2003. The goal of US-SCAP was to understand the treatment of patients with schizophrenia in usual care settings. Briefly, participants were enrolled from 6 regional sites (California, Colorado, Connecticut, Florida, Maryland, and North Carolina) and diverse systems of care, including community mental health centers, university health care systems, the Department of Veterans Affairs Health Services (VA), and community and state hospitals. Participants were diagnosed with schizophrenia, or schizoaffective or schizophreniform disorder, based on DSM-IV criteria and were at least 18 years of age. Individuals were excluded from the study if they were unable to provide informed consent or had participated in a clinical drug trial within 30 days prior to enrollment. Further details regarding US-SCAP are available elsewhere [15,27,28].

A total of 2327 participants were enrolled from 6 states and represented treatment in diverse systems of care including community mental health centers, university health care systems, the Department of Veterans Affairs Health Services (VA), and state hospitals. Institutional Review Board (IRB) approval was received at each regional site, and informed consent was received from all participants.

**Figure 1:**
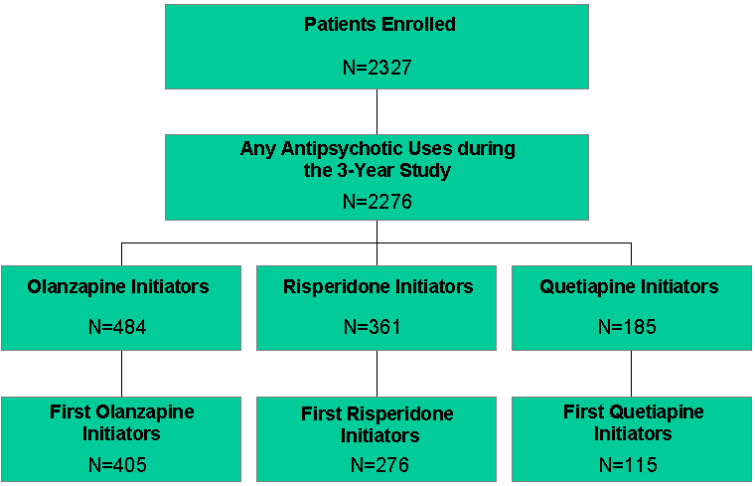
**Patient Disposition**. Disposition of patients initiated on any antipsychotic during the 3-year study and by antipsychotic drug.

The current study identified 2276 of the 2327 total participants in the study who used antipsychotics during the 3-year study period. Among those antipsychotic users, a total of 796 of olanzapine, risperidone, and quetiapine users who met the "initiator" criteria were identified (Figure 1). Participants were defined as initiators if they were not prescribed the index antipsychotic (olanzapine, risperidone, quetiapine) for at least 60 days prior to initiation and had at least 2 months of data available prior to and at least 12 months of data post initiation of the index antipsychotic. Inclusion of data for the 2 months prior to initiation allowed for the identification of variables on which the treatment groups differed at the time of initiation. Further, because this study aimed to assess use of antipsychotics over a 1-year period, availability of a 12-month follow-up period was necessary. This resulted in an initial analytical sample of 1030 (484, 361, 185 for olanzapine, risperidone, and quetiapine, respectively) participants. However, since some participants had more than 1 initiation on an index drug during the 3-year study, the final analytical sample (N = 796) included the participants' first initiation on the index medication in standard oral formulation resulting in 3 medication initiation groups: olanzapine (N = 405), quetiapine (N = 115), and risperidone (N = 276). Participants were not excluded from the analysis if they: (1) stopped their antipsychotic therapy as long as they completed the 1-year study, (2) initiated on another antipsychotic(s), and (3) augmented with other antipsychotics during the study.

### Outcome measures

Medical records provided information on all prescribed antipsychotics and were systematically abstracted by trained examiners for the 6 months prior to enrollment and for each 6-month interval thereafter. Patients were queried on the use of medications and other mental health resources outside those received at their regular treatment site. When this occurred, systematic efforts were made to abstract out-of-site medical records.

Antipsychotic medication costs were calculated on: (1) average daily cost of the index antipsychotic while patients were on the index antipsychotic, (2) average daily cost of the coprescribed antipsychotics while patients were on their index antipsychotic, (3) average daily cost of both the index and coprescribed antipsychotics while patients were on their index antipsychotic, and (4) total annual cost of antipsychotic medications prescribed in the year following initiation on the index antipsychotic. The first episode following initiation for each index medication was used to calculate the average daily cost of antipsychotic medication. The first episode following initiation was the total number of days from the date of initiation of the index drug to the first medication gap of more than 30 days. The total annual antipsychotic costs were the sum of all antipsychotic medication costs incurred during the 1-year post initiation of the index antipsychotic. Furthermore, to provide a more recent cost estimate, the daily costs and annual costs were also estimated using more recent (2004) antipsychotic drug prices.

The actual dose and frequency of each prescribed antipsychotic was identified for each patient for each day on the index antipsychotic and the coprescribed antipsychotic(s) during the 1 year following its initiation. This information was used to calculate the *average daily *costs and *the annual *costs. The average daily cost for each index antipsychotic and coprescribed antipsychotic (by type of coprescribed antipsychotic: typical or atypical) was calculated by multiplying the average daily dose of that antipsychotic incurred during the index treatment episode by the median milligram Average Wholesale Price (AWP) published for the study year of that antipsychotic, discounted by 15% for atypical antipsychotics to better reflect discount practices in the United States. Antipsychotic costs were based on AWP for 1998–1999, because these years constituted the midpoint in patients' enrollment in US-SCAP. For comparison purposes, a sensitivity analysis on the antipsychotic costs was also calculated based on 2004's AWP when the analysis for this study was conducted. We assume that AWP will reflect the inflation of costs, and no further adjustment based on inflation rate was made.

### Statistical methods

For the summary statistics, group comparisons were made on baseline characteristics using ANOVA for continuous variables and Mantel-Haenszel chi-square tests for categorical variables. In order to test the mean differences in average daily or total annual medication costs between the treatment groups, propensity score-adjusted bootstrap resampling method [29] was used with a set of *a priori *defined variables as adjustment. Patient demographic and available clinical characteristics (age, gender, race, illness duration, schizoaffective diagnosis, insurance, comorbid substance use disorder, psychiatric hospitalization at initiation, time elapsed between medication initiation and beginning of the study, and enrollment site) was included in the logistic models to calculate the propensity score for each patient. Propensity score-adjusted bootstrapping method analyzes mean differences of costs or days on medication by first calculating the logit score for each patient based on the above adjustments. There were 5 bins of logit scores created then for each treatment group. Bootstrap resampling method was then performed by randomly selecting an equal size of sample from each of the 10 bins (5 bins for each treatment) into 1 group and calculating the total cost difference between the 2 treatment groups compared. After 1000 times of iteration, the 1000 data points generated are tested using 2-tailed *p*-values. The same statistical approach was used to compare the average daily and total annual antipsychotic medication costs estimated using the 2004 drug prices. All statistical tests were 2-tailed, and significance was set at alpha of 0.05. SAS version 8.2 was used to perform all statistical analyses, and statistical significance was at a 2-sided alpha level of 0.05.

## Results

### Patient characteristics

Among the 2327 patients enrolled in this naturalistic, observational study, a total of 796 patients met inclusion criteria in this initiator's analysis: 405 were initiated on olanzapine, 276 on risperidone, and 115 on quetiapine (Figure 1). The average age for the patients in this study was 41.0 ± 11.0 years, and the average age of illness onset was 19.9 ± 8.8 years. The majority of the patients were male (57.3%) and had Medicaid and/or Medicare insurance coverage (82.1%). At the time of initiation on the index antipsychotic, the 3 treatment groups did not significantly differ on age, ethnicity, diagnosis of schizoaffective disorder, or uninsured status (Table 1). The groups, however, differed significantly (p < .05) on gender, substance use, and hospitalization status at the time of initiation. The quetiapine-initiated group was more likely to be female, of shorter illness duration, and lower likelihood of comorbid diagnosis of substance use disorder. The risperidone-initiated patients were more likely to be initiated on the drug during a psychiatric hospitalization, and the olanzapine-initiated group had longer illness duration and more patients with veteran status. As noted earlier, these baseline characteristics were included as covariates in the statistical models used to analyze cost differences between the groups.

**Table 1:** Comparison of baseline demographics and clinical characteristics of the study patients.

**Variables**	**OLZ** **(N = 405)**	**RIS** **(N = 276)**	**QUE** **(N = 115)**	**p-value**
Age at Baseline	41.8(10.5)	40.4(12.1)	39.62(10.9)	0.0826
Age of Illness Onset	19.5(8.3)	20.0(9.2)	21.0(9.9)	0.2516
Gender (% Male)	61.7%(n = 250)	54.7%(n = 151)	47.8%(n = 55)	0.0165

Race (%)				0.2584
White	46.7%(n = 189)	45.3%(n = 125)	53.9%(n = 62)	
Black	41.2%(n = 167)	36.6%(n = 101)	33.9%(n = 39)	
Hispanic	8.9%(n = 36)	13.8%(n = 38)	8.7%(n = 10)	
Others	3.2%(n = 13)	4.4%(n = 12)	3.5%(n = 4)	

Insurance (%)				0.5837
Medicaid/Medicare	83.0%(n = 327)	80.2%(n = 218)	83.9%(n = 94)	
Champus/VA	6.8%(n = 27)	5.2%(n = 14)	2.7%(n = 3)	
Private/HMO	2.3%(n = 9)	4.0%(n = 11)	2.7%(n = 3)	
Other Insurance	1.0%(n = 4)	1.5%(n = 4)	1.8%(n = 2)	
No Insurance	6.8%(n = 27)	9.2%(n = 25)	8.9%(n = 10)	

Marriage Status (% single)	63.0%(n = 254)	57.8%(n = 129)	61.7%(n = 71)	0.3885
Education (% High School or Less)	71.6%(n = 287)	63.9%(n = 175)	67.3%(n = 76)	0.1046

Comorbidity				
Diagnosis of Substance use (%)	30.9%(n = 125)	29.4%(n = 81)	19.1%(n = 22)	0.0467
Diagnosis of Mental Retardation (%)	7.6%(n = 31)	6.2%(n = 17)	8.7%(n = 10)	0.626
Diagnosis of Personality (%)	18.3%(n = 74)	13.0%(n = 36)	20.9%(n = 24)	0.0925
Diagnosis of Borderline (%)	2.7%(n = 11)	2.9%(n = 8)	5.2%(n = 6)	0.3827
Diagnosis of Mental Retardation or Borderline (%)	10.4%(n = 42)	8.3%(n = 23)	13.0%(n = 15)	0.3529

Schizoaffective disorder diagnosis at enrollment (%)	39.6%(n = 132)	33.3%(n = 92)	40%(n = 46)	0.3239

Hospitalization Status on Initiation day (%)	12.8%(n = 52)	22.1%(n = 61)	13.9%(n = 16)	0.0043

MADRS Score at Enrollment	15.6(10.3)	15.5(11.4)	16.3(11.4)	0.8006
PANSS Total at Enrollment	73.2(18.4)	72.9(18.9)	73.6(17.74)	0.9451
Total days on index drug post 1 year after initiation	280(121)	260(134)	239(136)	0.0048
Total days CONTINUOUSLY on index drug post 1-year after initiation	272(129)	250(1431)	225(147)	0.0023

### Cost of all antipsychotic medications

#### Annual antipsychotic medication costs

The cost of an antipsychotic medication is affected by its dose and the duration of treatment with the respective drug. For the 3 atypicals used in the study, the average (median) dose was 13.9 (10.0) for olanzapine, 330 (395) for quetiapine, and 4.3 (3.9) for risperidone. The average daily doses in mg/day of the index atypical antipsychotics during the 1-year post initiation were within the package insert guidelines.

In the 1-year post initiation, the olanzapine group had an average of 280 days on olanzapine (Table 2) and a medication acquisition cost of $3005 (Table 3) compared to 260 days on risperidone with medication acquisition costs of $1878 (p < .01) and 239 days on quetiapine with medication acquisition costs of $1881 (p < .01). During the same 1-year period, the quetiapine-initiated group incurred significantly higher costs from the use of other antipsychotics ($3439) compared to the risperidone ($1936, p < .01) and the olanzapine treatment groups ($1530, p < .01). As a result, the quetiapine-initiated group incurred significantly higher annual total cost of all antipsychotic medications ($5320) compared to olanzapine ($4536, p < .01) or risperidone ($3813, p < .01) (Table 3). The lower cost of the olanzapine group and the higher cost of the quetiapine group appear to be largely driven by longer days of concurrent first-generation antipsychotics in the olanzapine-treated patients (i.e., olanzapine: 105 days, risperidone: 79.3 days, quetiapine: 69.4 days, with a reverse pattern being true for cotreatment with atypicals (i.e., olanzapine: 36.1 days, risperidone: 49.4 days, quetiapine: 110.6 days).

**Table 2:** Total days on index drugs and concomitant antipsychotics.

**Concurrent drug class with index drug**	**OLZ** **(N = 405)**	**RIS** **(N = 276)**	**QUE** **(N = 115)**	p-value** (OLZ vs QUE)	p-value** (OLZ vs RIS)	p-value** (RIS vs QUE)
Total days on index drug post 1 year after initiation	280	260	239	0.0028	0.0435	0.0557
First episode of continuously on index drug	272	250	225	0.0023	0.0908	0.0162
Total concurrent days on other antipsychotics*						
on other atypicals	36.1	49.4	110.6	< 0.0001	0.0742	0.0001
on typicals	105.1	79.3	69.4	0.0107	0.0119	0.4723
on all anti-psychotics	136.6	120.3	160.2	0.1106	0.1378	0.0109
Percent of patients with >= 30 days of concurrent use						
on other atypicals	18.5%	25.4%	52.2%	< 0.0001	0.0322	< 0.0001
on typicals	48.2%	38.4%	33.0%	0.0041	0.0120	0.3171
on all anti-psychotics	61.7%	55.1%	72.2%	0.0396	0.0831	0.0017

* Total concurrent days of other antipsychotics while on the first episode of the index drug.

** Adjusted with comorbidity, age, race, gender, hospitalization status at initiation, schizoaffective diagnosis and insurance, site, time to initiation.

**Table 3:** Total costs of index drugs and other antipsychotics in the post 1-year total cost.

**Costs**	**OLZ** **(N = 405)**	**RIS** **(N = 276)**	**QUE** **(N = 115)**	p-value* (OLZ vs Que)	p-value* (OLZ vs RIS)	p-value* (RIS vs QUE)
Atypicals other index drug	868.04(1977.74)	1461.36(2615.2)	2938.38(3557.54)	< 0.001	0.016	< 0.001
Typicals	662.2(1343.21)	474.18(936.99)	500.81(995.88)	0.30	0.034	0.82
All other antipsychotics	1530.23(2255.42)	1935.54(2703.68)	3439.18(3614.58)	< 0.001	< 0.001	< 0.001
Index drug	3005.34(2163.62)	1877.92(1514.38)	1880.81(1766.23)	0.002	0.002	0.12
All antipsychotics including index drug	4535.57(3011.42)	3813.47(2890.79)	5320(3997.02)	0.002	0.004	0.002

* From 1000 iterations of bootstrap resampling.

#### Average daily costs

The average daily cost of antipsychotics was calculated while patients were on the first episode of their index medication in the 1 year following its initiation. Almost all index medication days on olanzapine, risperidone, and quetiapine occurred in the first episode (97%, 96%, and 94%, respectively, Table 2). The average daily cost of the index antipsychotic medication alone (Table 4) was the highest for olanzapine ($10.08), followed by risperidone ($6.74) and quetiapine ($6.63). Lower total average daily costs was observed in risperidone than olanzapine or quetiapine. However, quetiapine-initiated patients were significantly more likely to be coprescribed other atypical antipsychotics (i.e., 110.6 days/year) than risperidone-initiated patients (49.4 days/year, p < .01) or olanzapine-initiated patients (36.1 days/year, p < .01) (Table 2), costing an extra $7.44 per day on the coprescribed atypicals for the quetiapine-initiated patient, $3.15 for the risperidone-initiated patients (p < .01), and $2.02 for olanzapine-initiated patients (p < .01) (Table 4). In addition to concurrent use of other atypicals, there was also concurrent use of typical antipsychotics. The average daily cost of the coprescribed typical and atypical antipsychotics was significantly higher for quetiapine ($8.70) compared to risperidone ($4.30, p < .01) or olanzapine ($3.82, p < .01) (Table 4). This cost was driven mainly by costs of coprescribed atypical antipsychotics. The total average daily antipsychotic cost – of the index antipsychotic plus the coprescribed antipsychotic medications (typical and/or atypical) – was highest for quetiapine ($15.33), followed by olanzapine ($13.90, p < .01) and risperidone ($11.04, p < .01) (Table 4). Each dollar spent on the index atypical antipsychotic was accompanied by spending an additional $1.31 ($8.70/6.63) on concomitant antipsychotic medications for quetiapine compared to additional $0.64 ($4.30/6.74) for risperidone and additional $0.38 ($3.82/10.08) for olanzapine.

**Table 4:** Daily cost of index and concomitant antipsychotics used during the first episode of index medication.

**Costs**	**OLZ** **(N = 405)**	**RIS** **(N = 276)**	**QUE** **(N = 115)**	p-value* (OLZ vs Que)	p-value* (OLZ vs RIS)	p-value* (RIS vs QUE)
Concomitant atypicals	2.02(5.85)	3.15(6.85)	7.44(10.61)	< 0.001	0.17	< 0.001
Concomitant typicals	1.79(4.08)	1.15(2.44)	1.26(2.96)	0.220	0.004	0.850
Concomitant antipsychotics	3.82(6.88)	4.30(7.07)	8.70(10.84)	< 0.001	< 0.001	< 0.001
Index Drug	10.08(5.4)	6.74(3.81)	6.63(4.55)	0.002	0.002	0.140
All antipsychotics	13.9(8.97)	11.04(7.93)	15.33(11.71)	0.012	0.002	< 0.001

* From 1000 iterations of bootstrap resampling.

**Figure 2:**
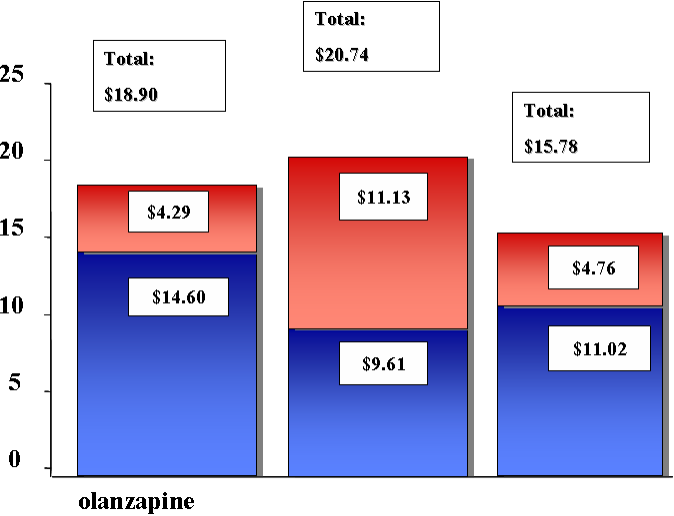
**Projected Total Daily Cost of All Antipsychotics Based on the Median 2004 AWP**. Projected total daily cost in U.S. dollars of all antipsychotics for patients initiated on olanzapine, quetiapine, and risperidone, using median 2004 average wholesales prices.

Using the median 2004 AWP to estimate the average daily cost of all antipsychotics generated a similar pattern to that obtained using 1998–1999 costs above. The estimated 2004 daily costs were $20.74, $18.90, and $15.78 per day for quetiapine, olanzapine, and risperidone, respectively with coprescribed antipsychotics being significantly higher for quetiapine ($11.13) compared to olanzapine ($4.29, p < .01) or risperidone ($4.76, p < .01) (Figure 2).

## Discussion

This study estimated total antipsychotic medication costs in the usual care of patients with schizophrenia treated at various health care settings in the United States when initiated on 1 of 3 commonly used atypical antipsychotics – olanzapine, risperidone, and quetiapine. The 2 approaches used to calculate the costs were the *average daily *cost while the patient is on the initiated index antipsychotic and the *total annual *antipsychotic medication costs, reflecting intent-to-treat approach. The average daily cost on the index atypical antipsychotic was significantly higher for olanzapine compared to risperidone and quetiapine groups. However, due to the presence of antipsychotic polypharmacy, each dollar spent on the index antipsychotic was accompanied by an additional spending on other antipsychotics, such that each dollar spent on quetiapine was associated with an additional $1.31 for concomitant antipsychotics, compared to an additional $0.64 for risperidone and an additional $0.38 for olanzapine. Thus, despite the significantly higher medication acquisition cost of olanzapine, the average daily antipsychotic medication cost was found to be significantly higher for quetiapine ($15.33) compared to olanzapine ($13.90) and risperidone ($11.04).

Similarly, the intent-to-treat annual antipsychotic medication cost for patients initiated on olanzapine ($4536) and risperidone ($3813) was found to be significantly lower than that for patients initiated on quetiapine ($5320), a reduction in medication cost of $786 for olanzapine and $1507 for risperidone. Importantly, these total antipsychotic cost savings for patients initiated on olanzapine compared to quetiapine were achieved despite longer treatment duration with olanzapine at a higher drug acquisition cost.

The primary cost driver of the expensive antipsychotic polypharmacy practice associated with quetiapine therapy appears to be the rates and duration of coprescribed atypical antipsychotic polypharmacy. As reported in our previous study [15], patients initiated on quetiapine had the highest rate and longest duration of antipsychotic polypharmacy among the 3 studied atypical antipsychotics, followed by risperidone and olanzapine, thus increasing the average daily and annual antipsychotic costs of treating patients with schizophrenia. Note that the higher antipsychotic medication costs associated with quetiapine therapy was achieved despite the relatively low dose of quetiapine used in this study. Low dose of quetiapine may explain its lower medication acquisition costs compared to olanzapine. In clinical practice, higher mean dose (620 mg) for quetiapine has been reported by Citrome and colleagues [30]. There is the possibility that quetiapine was dosed too low in this study to be effective for some patients leading to unnecessary costs of additional antipsychotics. At the same time, the 2-fold increase in quetiapine dose will considerably increase its acquisition costs and overall medication costs.

To our best knowledge, this is the first study to assess the cost of antipsychotic polypharmacy in the usual care of patients with schizophrenia demonstrating that this common practice adds a substantial treatment cost and may even double the medication cost of antipsychotic medication regimens. Current findings are consistent with previous research [19-21,31]. In a short-term, randomized, double-blind study of risperidone, placebo, and quetiapine for inpatients with schizophrenia [31], clinicians were allowed to augment with other antipsychotics for a 4-week duration following 2 weeks of monotherapy. That study found the quetiapine group (mean dose 579.5 mg/day) to be significantly more likely augmented with another antipsychotic compared to risperidone, but without significant or meaningful clinical benefits. The mean cost of antipsychotic polypharmacy per randomized patient was almost twice as high for the quetiapine group. Findings of the present study, along with previous research on the prevalent use of antipsychotic polypharmacy [3,4,6] and its unproven benefits [32,33] in the treatment of schizophrenia, suggest that meaningful economic comparisons among atypical antipsychotics can only be achieved by incorporating the cost of antipsychotic polypharmacy in the total medication cost calculations. Findings also suggest the need for additional research to help clarify the underlying reasons for antipsychotic polypharmacy in the treatment of patients with schizophrenia in usual care.

Results of this study need to be evaluated in the context of its limitation. First, this was a nonrandomized, noninterventional, observational study; thus, treatment group selection bias cannot be eliminated despite our use of statistical adjustments – with the propensity score-adjusted bootstrapping method – for various available patient characteristics at the time of medication initiation. Second, this study focused on comparing costs associated with the use of antipsychotics.

The use of other psychotropics may vary among treatment groups and may contribute differentially to the total psychiatric medication costs for different treatment groups. Further studies on the use of all psychotropic medication and its associated costs will provide a better understanding of the costs associated with the use of various atypical antipsychotics in the treatment of schizophrenia. It should be noted that antipsychotics that were on generic such as clozapine or are soon on generic such as risperidone and ziprasidone will be cheaper than their brand names; therefore, the medication costs will be cheaper. In addition, clozapine is an important atypical antipsychotic medication in the treatment of schizophrenia. We decided to only focus on polypharmacy costs associated with the treatment of 3 most commonly used atypical antipsychotics – olanzapine, quetiapine, and risperidone and, at the same time, included any medication costs incurred by clozapine when it was co-prescribed with any of the 3 study medications to ensure that the results and conclusions from this study on the 3 medications are not affected by the exclusion of clozapine as a comparative treatment group. Ziprasidone and aripiprazole were not included in this study due to lack of data for these 2 medications. Ziprasidone was introduced in the United States toward the end of US-SCAP, and aripiprazole was launched after US-SCAP completion.

Finally, this study is unable to explain why there was a greater propensity of olanzapine to be coprescribed with typical antipsychotics and of quetiapine to be coprescribed with atypicals. The US-SCAP did not assess the reasons for clinicians' medication choices, including the reasons for medication initiation or augmentation. Clinicians may prescribe antipsychotics for different reasons to patients with different illness profiles. As a result, the cost advantage of olanzapine over quetiapine may be the result of a biased pattern of prescribing unique to clinicians participating in this protocol. Fully understanding and providing measures for the reasons behind the medication prescription pattern will help better address the potential selection bias associated with an observational study. In this study, we adjusted patient's demographics, hospitalization, illness history, and other observable factors. Unobservable factors may still exist and may create imbalance between the treatment groups.

## Conclusion

Prevalent antipsychotic polypharmacy adds substantial cost to the treatment of schizophrenia with significant and meaningful differences in antipsychotic polypharmacy use patterns among atypical antipsychotics. Consistent with previous research, schizophrenia patients initiated with quetiapine appear more likely to receive antipsychotic polypharmacy leading to an increase in antipsychotic medication costs compared to olanzapine and risperidone-initiated patients. Current findings, based on treatment of schizophrenia patients in usual care settings across the United States, have important economic implications for formularies, health care insurers, and the mental health system, indicating that the extra costs of antipsychotic polypharmacy should be considered when evaluating the economic implications of treatment with any specific antipsychotic medication. The customary unilateral focus on the cost of the index antipsychotic medication acquisition alone does not accurately reflect the incurred total costs of antipsychotic medications. A better understanding of the concomitant antipsychotic costs provides for a more accurate portrayal of antipsychotic medication costs in the treatment of schizophrenia.

## Competing interests

This work was sponsored by Eli Lilly and Company. Baojin Zhu, Haya Ascher-Svanum, and Douglas Faries are full-time employees and minor stockholders of Eli Lilly and Company, Indianapolis, IN. Christoph U. Correll and John M. Kane are employees of The Zucker Hillside Hospital, Glen Oaks, NY.

## Authors' contributions

BZ helped develop the idea for the study, helped design the study, participated in the analysis, and participated in the writing of the manuscript. HA-S and DEF helped develop the idea for the study, helped design the study, and participated in the writing of the manuscript. CUC and JMK assisted in the writing of the manuscript. All authors read and approved the final manuscript.

## Pre-publication history

The pre-publication history for this paper can be accessed here:


